# Effect of Monthly, High-Dose, Long-Term Vitamin D on Lung Function: A Randomized Controlled Trial

**DOI:** 10.3390/nu9121353

**Published:** 2017-12-13

**Authors:** John D. Sluyter, Carlos A. Camargo, Debbie Waayer, Carlene M. M. Lawes, Les Toop, Kay-Tee Khaw, Robert Scragg

**Affiliations:** 1 School of Population Health, University of Auckland, Auckland 1072, New Zealand; d.waayer@auckland.ac.nz (D.W.); carlene.lawes@waitematadhb.govt.nz (C.M.M.L.); r.scragg@auckland.ac.nz (R.S.); 2 Department of Emergency Medicine, Massachusetts General Hospital, Harvard Medical School, Boston, MA 02114, USA; ccamargo@partners.org; 3 Department of General Practice, University of Otago, Christchurch 8011, New Zealand; les.toop@otago.ac.nz; 4 Department of Public Health and Primary Care, University of Cambridge, Cambridge CB3 OEQ, UK; kk101@medschl.cam.ac.uk

**Keywords:** vitamin D, lung function, forced expiratory volume in 1 s, spirometry, randomized controlled trial

## Abstract

Although observational studies suggest positive vitamin D-lung function associations, randomized trials are inconsistent. We examined effects of vitamin D supplementation on lung function. We recruited 442 adults (50–84 years, 58% male) into a randomized, double-blinded, placebo-controlled trial. Participants received, for 1.1 years (median; range = 0.9–1.5 years), either (1) vitamin D_3_ 200,000 IU, followed by monthly 100,000 IU doses (*n* = 226); or (2) placebo monthly (*n* = 216). At baseline and follow-up, spirometry yielded forced expiratory volume in 1 s (FEV1; primary outcome). Mean (standard deviation) 25-hydroxyvitamin D increased from 61 (24) nmol/L at baseline to 119 (45) nmol/L at follow-up in the vitamin D group, but was unchanged in the placebo group. There were no significant lung function improvements (vitamin D versus placebo) in the total sample, vitamin D-deficient participants or asthma/chronic obstructive pulmonary disease (COPD) participants. However, among ever-smokers (*n* = 217), the mean (95% confidence interval) FEV1 increase in the vitamin D versus placebo was 57 (4, 109) mL (*p* = 0.03). FEV1 increases were larger among vitamin D-deficient ever-smokers (*n* = 54): 122 (8, 236) mL (*p* = 0.04). FEV1 improvements were largest among ever-smokers with asthma/COPD (*n* = 60): 160 (53, 268) mL (*p* = 0.004). Thus, vitamin D supplementation did not improve lung function among everyone, but benefited ever-smokers, especially those with vitamin D deficiency or asthma/COPD.

## 1. Introduction

Population-based observational studies have found that low serum 25-hyroxyvitamin D (25(OH)D) concentration is associated with poor lung function [1,2]. However, the observational design prevents one from knowing whether these relationships are causal, or whether they could be reversed by increasing 25(OH)D. To investigate the causality and reversibility of these associations, randomized controlled trials (RCTs) of vitamin D supplementation are required.

A limited number of RCTs have investigated the effect of vitamin D supplementation on lung function in adults [3,4,5,6,7,8,9,10]. However, vitamin D efficacy remains unclear, due to the conflicting findings of these trials: some reported beneficial changes [7,8,11,12], others found no effects [3,4,10], and another reported mixed results [9]. Most of these studies had relatively small sample sizes (*n* ≤ 130) [3,4,7,8,9] and short follow-up periods (<1 year), which limited their ability to assess long-term efficacy [3,4,6,7,8,9]. Nearly all of these studies were restricted to patients with respiratory conditions such as asthma [6,8,9,10] or chronic obstructive pulmonary disease (COPD) [4,5,7]. However, vitamin D trials should include other groups of people, too, in order to study the role of vitamin D supplementation on lung health in general [13]. Trials should also investigate vitamin D-deficient people and smokers, as vitamin D supplementation could potentially be more effective in these people. This is because non-linear relationships between 25(OH)D and health outcomes suggest that adverse effects associated with low vitamin D status are greatest in vitamin D-deficient people [14,15,16], while observational studies suggest that the relationship between 25(OH)D and lung function could be stronger in smokers than in non-smokers [1,17,18,19].

Given the above knowledge gaps, we used an RCT design to investigate the effect of long-term (≥1 year on average), high-dose vitamin D supplementation on lung function in a population-based sample of >400 adults. We performed pre-specified subgroup analyses among participants who had vitamin D deficiency or asthma/COPD, or were smokers.

## 2. Material and Methods

### 2.1. Participants

The present study involved a pre-specified analysis of a sub-sample of participants in the ViDA (Vitamin D Assessment) study, who underwent follow-up measurements for ~1 year. The ViDA study was a randomized, double-blinded, placebo-controlled trial of the effect of vitamin D supplementation on health outcomes, with cardiovascular disease as the primary endpoint. Participants were identified mostly from patient lists of family practices, and the remainder from ethnic minority community groups, and we recruited them to our study by post and subsequent follow-up telephone calls. Inclusion criteria were men and women from these family practice registers and community groups, aged 50–84 years and resident in Auckland at recruitment. Exclusion criteria included: (1) diagnosis of a terminal illness and/or in hospice care; (2) intending to leave New Zealand during the follow-up period; (3) taking vitamin D supplements (including cod liver oil) of >600 IU daily if aged 50–70 years or >800 IU daily if aged 71–84 years; (4) history of renal stones, hypercalcemia, or medical conditions that can cause hypercalcemia; and (5) baseline serum calcium >2.50 mmol/L. Screening and baseline measurements took place at the School of Population Health (University of Auckland) between 2011 and 2012, with 5110 being randomized using computer generation to receive either vitamin D or placebo. Random assignment to one of the two treatment groups was made with random block sizes of 8, 10 or 12, within ethnic and 5-year age groups. As each participant became eligible for randomization, the next sequential treatment within their ethnic and age stratum was allocated. The randomization process was supervised by the study biostatistician to ensure that participants and staff who collected the data were blinded to allocation. Ethics approval was provided by the New Zealand Multi-region Ethics Committee (MEC/09/08/082). Written, informed consent was obtained from each participant. This study was registered with the Australian New Zealand Clinical Trials Registry (http://www.anzctr.org.au; ACTRN12611000402943). Full study-design details have been published elsewhere [20]. The results are reported according to Consolidated Standards of Reporting Trials (CONSORT) guidelines [21].

### 2.2. Vitamin D Intervention

Vitamin D_3_ (100,000 IU (2.5 mg)) or placebo softgel oral capsules, sourced from Tishcon Corporation (Westbury, NY, USA), were mailed to participants’ homes. Two capsules were sent in the first mail-out after randomization (that is, a 200,000 IU bolus, or placebo, at the start of the intervention period), followed by a monthly 100,000 IU (daily dose equivalent ~3300 IU/day) capsule of vitamin D_3_ (or placebo) throughout the remainder of the trial. To achieve masking, the vitamin D and placebo capsules were identical in appearance.

### 2.3. Non-Lung Function Measures

All measurements were carried out by trained staff using a standardized protocol. Questionnaires administered by interviewers were used to collect data on age, sex, ethnicity (defined by self-identification), smoking, sun exposure, history of asthma (including the Asthma Control Test (ACT) [22]), use of vitamin D supplements, and medications for asthma, chronic bronchitis or emphysema. An ACT score of ≥20 denoted well-controlled asthma [23]. An ever-smoker was defined as a current or former smoker. Participants were also asked the number of years since quitting (for ex-smokers) and the number of cigarettes smoked per day (for current smokers). Lung-related medications were determined from the questionnaire (described above) and national medicine-dispensing database. Without shoes and in light clothing, height (±0.1 cm) was measured with a stadiometer, and weight (±0.1 kg) with digital scales. Body mass index was calculated as weight (kg)/height (m^2^).

Blood samples were collected at baseline, and at 6 and 12 months of follow-up to measure calcium for hypercalcemia (corrected calcium > 10.4 mg/dL). Remaining plasma aliquots were stored frozen at −80 °C. Serum 25(OH)D (combining D_2_ and D_3_) concentration was measured in these aliquots (baseline and follow-up samples were measured in the same batch for each participant) by liquid chromatography-tandem mass spectrometry (ABSciex API 4000, Framingham, MA, USA) at a laboratory participating in the Vitamin D External Quality Assessment Scheme (DEQAS) program (www.deqas.org).

### 2.4. Lung Function Measures

Lung function was assessed with a KoKo Trek spirometer (nSpire Health, Longmont, CO, USA) in a seated position. All published recommendations for spirometry measurement [24] were adhered to, except attaining three acceptable measures from a maximum of 8 efforts. In our study, which was ancillary to a much larger trial of 5110 participants who all had the same standard baseline assessment, only three efforts were performed due to time constraints and to avoid exhaustion in elderly participants. Participants were educated about the spirometric technique verbally and via demonstration, and while watching a clock on the computer screen were encouraged to inhale and exhale as maximally and forcibly as possible for at least 6 s. Maximum values of forced expiratory volume in 1 s (FEV1; in mL), forced vital capacity (FVC; in mL) and FEV1 as a percentage of FVC (FEV1/FVC) from three efforts (not necessarily from the same effort) were used for analyses. FEV1 was the primary outcome [12], with FVC and FEV1/FVC as secondary outcomes. The quality of the spirometry measurements was graded (from A to F) and classified into two groups: measurements with grades A or B (representing higher-quality measurements) and those with grades C to F (representing lower-quality measurements). A and B grades included participants with acceptable spirometry, with at least two measurements out of three within 150 mL of one another and exhalation time of more than 6 s [24].

A FEV1/FVC value of <70% was used to define COPD, as recommended by the 2017 Global Initiative on Obstructive Lung Disease (GOLD) classification of COPD [25]. COPD severity was graded using GOLD stages, which are based on percentage of predicted FEV1 values (where predicted FEV1 values were calculated from published equations [26]): stage 1 (mild): ≥80%; stage 2 (moderate): 50–79%; stage 3 (severe): 30–49%; and stage 4 (very severe): <30% [25]. These equations were also used to calculate spirometric *z*-scores [26].

### 2.5. Statistical Analysis

Data were analysed using SAS version 9.3 (SAS Institute, Cary, NC, USA). On an intention-to-treat basis, PROC MIXED general linear mixed models were used to assess the effect of vitamin D supplementation (exposure) on the outcomes, 25(OH)D and lung function parameters (adjusted for age, sex, ethnicity and height), with repeated time incorporated using an unstructured correlation structure. This analysis method handles missing data by fitting a statistical model over all available observations without introducing bias. Potential three-way interactions between spirometry grade acceptability (grades A or B versus other grades), treatment group and time were examined, but as they were not statistically significant, we did not report results by spirometry grade acceptability.

Deseasonalised (season-adjusted) baseline 25(OH)D levels were calculated for each participant from the mid-point between the estimated maximum and minimum 25(OH)D levels over a calendar year, from their individual measured baseline 25(OH)D and date of blood collection, using a sinusoidal model with parameters derived from baseline values for all participants in the main ViDA study [27]. Vitamin D deficiency was defined as having a deseasonalised 25(OH)D of <50 nmol/L [27].

Aside from performing analysis among all eligible participants (total sample), we decided a priori to conduct subgroup analyses among participants with baseline vitamin D deficiency, asthma/COPD, and a smoking history (ever-smokers), given the abovementioned rationale for these. These analyses included combinations of these subgroups to explore potentially cumulative effects. Due to small sample sizes, especially in these mixed-subgroup analyses, we combined asthma cases with COPD cases and ex-smokers with current smokers. In further analyses, we examined three-way interactions—between smoking status (ever-smoker or not), treatment group, and time—to test whether the effects of vitamin D were different in ever-smokers than in never-smokers.

The ViDA study was originally powered to detect a clinically relevant reduction in cardiovascular events (primary outcome), as described elsewhere [20]. For the current sub-study, with a standard deviation of FEV1 change of 180 mL, 80% power and at the 5% significance level (two-tailed), the minimum detectable differences in FEV1 were about 48 mL in the total sample, 89 mL in all vitamin D-deficient people, 96 mL in everyone with asthma/COPD, and 69 mL in all ever-smokers.

Correlations between changes in observed 25(OH)D and changes in lung function parameters were summarized with Pearson correlation coefficients (*r*). Robust estimates (95% confidence intervals) of these correlation coefficients were calculated using 1000 bootstrap samples. *p*-values were not corrected to account for multiple hypothesis tests, as we did not want to miss any potentially important findings [28]. A two-sided *p* < 0.05 was considered statistically significant.

## 3. Results

**Figure 1:**
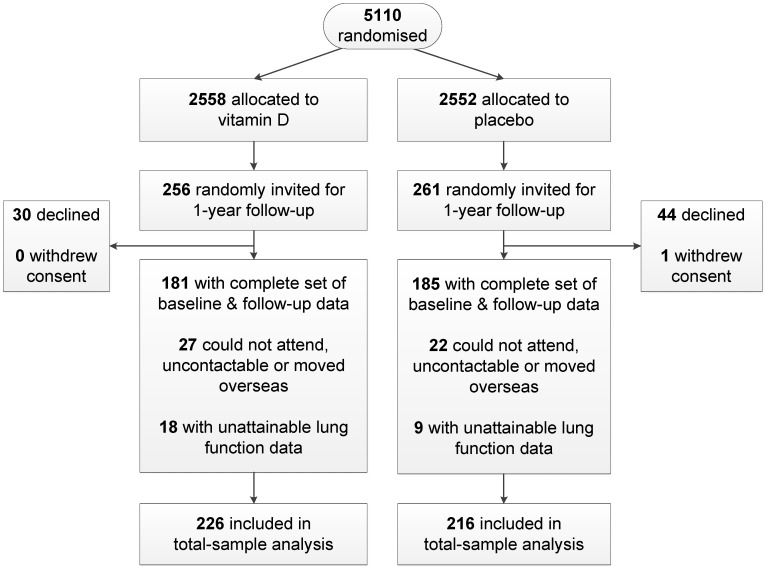
Flowchart showing the number of randomized participants who were excluded and those with a complete set of both baseline and 1-year follow-up measurements.

The study flowchart is shown in Figure 1. From the 5110 participants randomized in the main ViDA study, 517 (10%) were randomly selected and invited to partake in the current sub-study. Of these, 74 declined and 1 withdrew consent (data analysis prohibited), and were thus not included in subsequent analyses. Out of the remaining 442, a complete set of both baseline and 1-year follow-up measurements was available in 366 participants (83%). The 76 participants with missing data comprised 49 who did not attend the follow-up interview (could not attend, uncontactable or moved overseas) and 27 who had unobtainable spirometry data (could not obtain a reading). Per intention-to-treat, all 442 people were included in the total-sample analysis. The proportion of the total sample with missing follow-up data did not differ across the two treatment groups (*p* = 0.96, χ^2^ test). Further, baseline lung function of those with missing follow-up data did not differ from those without missing follow-up data (*p*-values ranging from 0.47 to 0.57; analysis of variance.)

Of the total sample, 226 received vitamin D and 216 received the placebo. Table 1 shows the baseline characteristics of these participants by treatment group. The follow-up period (randomization—follow-up) averaged 1.1 years (mean and median) and ranged from 0.9 to 1.5 years. The mean age was 65 years (range: 50–84 years), 58% were male and just over three-quarters were of European/Other ethnicity (with 96% having European ancestry). Fourteen percent had asthma (77% well-controlled—ATS score ≥ 20) and 17% had COPD (mostly mild or moderate—GOLD stages 1 and 2). Nearly 30% had a deseasonalised 25(OH)D of <50 nmol/L (vitamin D deficiency). Almost one-half (49%) had smoked (82% of whom were ex-smokers). Supplementary analyses (not tabulated) showed that ex-smokers had quit a median of 30 years earlier (interquartile range: 13 to 40 years), and the median number of cigarettes smoked per day among current smokers was approximately just under 10 (54% smoked ≤10 per day). Further supplementary analyses showed that the proportion of participants with spirometry grades A or B were similar in the vitamin D (30%) and placebo (28%) groups.

**Figure 2:**
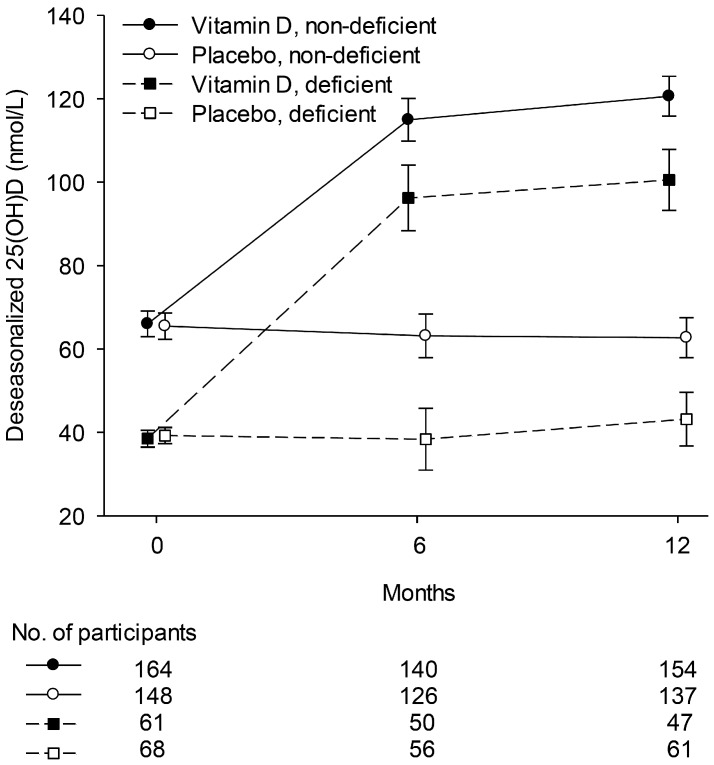
Line graph showing deseasonalised 25(OH)D concentration (mean ± 95% confidence intervals; in nmol/L) at baseline and follow-up (6 and 12 months) in the vitamin D and placebo groups, for non-vitamin D-deficient and vitamin D-deficient people. Baseline 25(OH)D was missing for 1 person (vitamin D group).

The deseasonalised 25(OH)D concentrations at baseline and follow-up visits by treatment group are illustrated in Figure 2. In the total sample, the change (95% confidence interval) from baseline in the vitamin D group compared to placebo at 6 and 12 months follow-up, respectively, was +51 (45, 58) and +57 (51, 64) nmol/L (*p* < 0.001). No cases of hypercalcemia were detected.

FEV1 at baseline and follow-up by intervention group is shown in Table 2. All effects were in the positive direction for the vitamin D supplemented group compared to placebo. The effects were non-significant in the total (*n* = 442), vitamin D-deficient (*n* = 130) and asthma/COPD (*n* = 113) samples. This was true, too, among participants with both asthma/COPD and vitamin D-deficiency, despite the effect being large (109 mL) and borderline significant (*p* = 0.08). Conversely, among all ever-smokers, FEV1 significantly increased in the vitamin D group with respect to placebo (*p* = 0.03), with a mean (95% confidence interval) change of 57 (4, 109) mL. This effect more than doubled when restricted to ever-smokers who also had vitamin D deficiency (β = 122 mL, *p* = 0.04) or asthma/COPD (β = 160 mL, *p* = 0.004). Similar patterns were observed when we modelled FEV1 as a *z*-score, with net (placebo-controlled) vitamin D effects of 0.13 (0.01, 0.24; *p* = 0.03) and 0.35 (0.11, 0.59; *p* = 0.005) *z*-scores among all ever-smokers and ever-smokers with asthma/COPD, respectively (Table S1).

When these analyses were repeated with FVC as the response variable (Table 3), all effects were smaller (compared to FEV1 effects). The effect of vitamin D compared to placebo was minimal and non-significant (*p* > 0.05) in the total sample. Across subgroup samples, the effect was larger (in nearly all cases) and consistently in the positive direction, although still not statistically significant. Similar patterns were observed with FVC as a *z*-score (Table S2).

When these analyses were repeated with FEV1/FVC as the dependent variable, all effects (vitamin D compared to placebo) were in the positive direction (Table 4). The effect among all ever-smokers was 1.1% (*p* = 0.05) and almost tripled when confined to ever-smokers who also had asthma/COPD (β = 3.0%, *p* = 0.01). Similarly, all FEV1/FVC *z*-score effects were in the positive direction, with the largest being among ever-smokers with asthma/COPD (β = 0.37, *p* = 0.01; Table S3).

Further analysis showed that vitamin D (with respect to placebo) significantly improved FEV1 more in ever-smokers than in never-smokers (*p* = 0.02 for three-way interaction between smoking, treatment group, and time). We confirmed this interaction when we restricted this analysis to people with vitamin D deficiency (*p* = 0.048) and asthma/COPD (*p* = 0.0005).

Correlations between changes in observed 25(OH)D concentration and changes in lung function measures are shown in supplementary Table S4. All FEV1 and FEV1/FVC correlations were in the positive direction. Among ever-smokers and their subgroups, 25(OH)D change was positively correlated (*r* = 0.17 to 0.34) with change in FEV1, which mirrors the FEV1 increases in these samples shown in Table 2.

## 4. Discussion

This randomized, double-blinded, placebo-controlled trial showed that monthly, high-dose vitamin D supplementation for just over 1 year did not affect lung function in the total sample, nor in subgroups defined by either vitamin D deficiency (<50 nmol/L) or having asthma/COPD. However, vitamin D supplementation did result in larger, statistically significant increases in FEV1 and FEV1 *z*-score among ever-smokers, especially those with vitamin D deficiency (FEV1 only) or asthma/COPD.

To our knowledge, this is the first study to show that vitamin D supplementation (compared to placebo) increases FEV1 and FEV1 *z*-score in ever-smokers. The restriction of this effect to ever-smokers only is consistent with observational research, which has shown stronger 25(OH)D-lung function associations among smokers [1,17,18,19] and a stronger smoking-FEV1 relationship in vitamin D-deficient people [2]. Taken together, these findings suggest that vitamin D supplementation may mitigate smoking-associated lung function damage, although smoking avoidance and cessation remain paramount for preserving lung health.

Smoking decreases the production of 1,25-dihydroxyvitamin D in lung epithelial cells [29] and may affect expression levels of the vitamin D receptor [30]. Smoking-related lung destruction is partly mediated through inflammation, oxidative stress, and increased proteases [31,32], and these pathophysiological changes may persist even after smoking cessation [33]. However, vitamin D could mitigate these processes [34,35,36]. Further, there is increased activity of these processes in asthma [37] and COPD [31]. Collectively, these observations could explain our finding that vitamin D effects were limited to ever-smokers and were the largest in ever-smokers with asthma/COPD.

The effects of vitamin D among all asthma/COPD participants were non-significant (although in the positive direction), which concurs with some prior RCTs of patients with asthma [9,10] or COPD [4,5]. We build on these past trials both by showing that the effects on asthma/COPD participants were stronger in ever-smokers (Table 2 and Table 3) and because only one of these studies used the same dosing regimen we administered (monthly ≥100,000 IU dosing for ≥1 year) [5]. That study differed from ours in that it comprised largely men (80%) with both mostly severe or very severe COPD and a history of recent exacerbations [5]; in contrast, our COPD cases were primarily mild or moderate, and were combined with predominantly well-controlled asthma cases.

The intervention effect for FEV1 as a percentage of the average lung function parameter value in the vitamin D group (both in Table 2) was modest (3%) among all ever-smokers (57 mL as a percentage of 2241 mL), larger (5%) among vitamin D-deficient ever-smokers (122 mL as a percentage of 2348 mL), and sizeable (10%) among asthma/COPD ever-smokers (160 mL as a percentage of 1538 mL). A change in FEV1 of at least 100 mL is considered to be clinically relevant [38,39], suggesting that the net vitamin D effects on FEV1 among vitamin D-deficient ever-smokers (122 mL) and asthma/COPD ever-smokers (160 mL) in our study (Table 2) represent, by definition, clinically meaningful improvements. Our FEV1 *z*-score results, which account for spirometric influences of demographics and height using a different statistical approach, provide further support of a benefit, with net vitamin D effects of 0.13 among all ever-smokers and 0.35 among ever-smokers with asthma/COPD (Table S1). Because there is a paucity of information on the size of the association between FEV1 *z*-scores and health outcomes [40,41], more such research is required to quantify the clinical impact of these *z*-score results. As for FEV1/FVC, the effects on this parameter were meaningful: given that FEV1/FVC declines by ~0.2% per year [26], the net vitamin D effects (increases) in FEV1/FVC of 1.1% among all ever-smokers and 3.0% among ever-smokers with asthma/COPD (Table 4) would correspond to changes that typically occur over 5.5 years and 15 years, respectively. Finally, as vitamin D effects on FEV1 were markedly more positive when analyses in asthma/COPD or vitamin D-deficient participants were restricted to ever-smokers than when they were not, this suggests that future RCTs of asthma, COPD or vitamin D-deficient people should carry out subgroup analyses among smokers to capture a potential difference in treatment effects.

Our study sample was population-based, as the vast majority of New Zealand residents (94%) are registered with family practices [42]. This augments the external validity of our findings. Regarding limitations, the missingness of the intention-to-treat sample (Figure 1) renders our study findings prone to selection bias. However, as mentioned, this missingness did not differ across the treatment groups, and did not predict baseline lung function. The data analyst was not blinded to the treatment group, although we did include pre-specified analyses in our statistical analysis plan (mentioned above). The equations used to calculate our *z*-score results [26] may have limited applicability to our Maori and Pacific participants, as these ethnic groups were not included in the data that these *z*-scores are based on. A longer follow-up period may have allowed us to better evaluate the long-term efficacy of the intervention. Although our total sample size was large, relative to previous RCTs of vitamin D and lung function [3,4,5,6,7,8,9,10], our statistical power was limited (particularly for the subgroup analyses), which may explain why at least some treatment effects were not statistically significant. Finally, the multiplicity of statistical tests we performed raises the possibility that at least some of our significant findings may have been due to chance. However, we observed positive dose-response relationships between change in 25(OH)D and change in lung function (Table S4), which supports a true effect (biological gradient). Further support includes the fact that, as reported, the FEV1 and FEV1 *z*-score treatment effects were consistent with observational research, biologically plausible, and were all unidirectional (across samples; Table 2). Also, if study conclusions are based on the primary outcome (FEV1) results only, far fewer comparisons are involved.

Our analyses were based on subsamples of an RCT (Figure 1). Although a limitation, we do not expect there to be marked, systematic differences in baseline participant characteristics, for the following reasons: Firstly, the selection of our total analysis sample from the main ViDA study was random. Second, as everybody was randomized in the same way, the selection of subgroups from the total sample should not differ across treatment groups. Third, since the analyses controlled for age, sex, ethnicity and height, effects of any imbalances in these demographic variables would have been minimized. Fourth, stratifying the study randomization by our subgroup variables (vitamin D deficiency, asthma/COPD and ever-smokers) could have reduced any baseline imbalances within these subgroups [43]. Although we did not do this, this effect would have been partially captured, as we stratified randomization by age and ethnicity, which are associated with these subgroup variables [44,45]. Further, some have proposed that stratification of randomization is not required for pre-specified subgroup analyses (such as ours) [46].

In summary, monthly high-dose vitamin D supplementation over an average of 1.1 years, which increased serum 25(OH)D concentration by >50 nmol/L with respect to placebo, did not improve lung function in the overall study population. In subgroup analyses, we found that vitamin D supplementation improved lung function (FEV1 and FEV *z*-score) in ever-smokers, particularly those with vitamin D deficiency (FEV1 only) or asthma/COPD. We encourage similar RCTs in smokers to assess the efficacy of different dosing regimens (e.g., daily or weekly supplementation). Additional RCTs are needed to investigate whether the observed beneficial effects translate into improvements in lung function-related health, such as improved asthma/COPD control.

## Figures and Tables

**Table 1:** Baseline characteristics of all participants across treatment groups.

Variable	Vitamin D	Placebo
*n*	226	216
Days from randomization to follow-up ^1^	401 ± 29	402 ± 30
Age (years) ^1^	64.6 ± 8.4	65.4 ± 9.0
Male sex (*n* (%))	140 (62)	117 (54)
Ethnicity		
European/Other (*n* (%))	172 (76)	169 (78)
Maori (*n* (%))	15 (7)	13 (6)
Pacific (*n* (%))	22 (10)	16 (7)
South Asian (*n* (%))	17 (8)	18 (8)
Asthma (*n* (%))	28 (12)	36 (17)
ACT score, median ± IQR	23.5 ± 4.5	21.5 ± 5.5
COPD (*n* (%))	40 (18)	37 (17)
GOLD stage 1 (*n* (%))	15 (7)	14 (6)
GOLD stage 2 (*n* (%))	19 (8)	18 (8)
GOLD stage 3 (*n* (%))	4 (2)	2 (1)
GOLD stage 4 (*n* (%))	2 (1)	3 (1)
Body mass index (kg/m^2^) ^1^	28.6 ± 5.2	28.5 ± 4.9
25-hydroxyvitamin D		
Observed ^1^	61.5 ± 24.4	61.4 ± 23.7
Deseasonalised ^1^	66.0 ± 23.7	65.5 ± 23.3
Deseasonalised < 50 nmol/L (*n* (%))	61 (27)	68 (31)
Lung function medication (*n* (%))	21 (9)	27 (13)
On vitamin D supplements at baseline (*n* (%))	27 (12)	22 (10)
Smoking		
Never-smoker (*n* (%))	122 (54)	103 (48)
Ex-smoker (*n* (%))	83 (37)	95 (44)
Current smoker (*n* (%))	21 (9)	18 (8)
Sun exposure (hours/day)		
<1 (*n* (%))	34 (15)	29 (13)
1–2 (*n* (%))	113 (50)	118 (55)
>2 (*n* (%))	79 (35)	69 (32)

Abbreviations: ACT = Asthma Control Test; COPD = chronic obstructive pulmonary disease; GOLD = 2017 Global Initiative on Obstructive Lung Disease classification; IQR = interquartile range. ^1^ Values are mean ± standard deviation.

**Table 2:** Forced expiratory volume in 1 s (mL) at baseline and follow-up (adjusted for age, sex, ethnicity and height) by treatment group.

Sample	*n*		Mean (Standard Deviation)				Change from Baseline, Vitamin D Minus Placebo	
	Vitamin D Group	Placebo Group	Vitamin D Group		Placebo Group			
			Baseline	Follow-Up	Baseline	Follow-Up	Mean (95% CI)	*p*-Value
Total	226	216	2242 (684)	2313 (687)	2370 (754)	2325 (737)	16 (−19, 51)	0.38
Vitamin D-deficient ^1^	61	68	2363 (597)	2363 (620)	2201 (777)	2162 (774)	39 (−28, 107)	0.25
Asthma/COPD	54	59	1869 (600)	1861 (623)	1951 (735)	1903 (691)	40 (−33, 112)	0.28
Vitamin D-deficient ^1^ + asthma/COPD	16	27	2023 (563)	2079 (574)	1914 (896)	1861 (844)	109 (−15, 233)	0.08
Ever-smoker	104	113	2241 (725)	2232 (750)	2262 (733)	2197 (693)	57 (4, 109)	0.03
Ever-smoker + vitamin D-deficient ^1^	26	28	2348 (641)	2378 (781)	1912 (616)	1821 (514)	122 (8, 236)	0.04
Ever-smoker + asthma/COPD	25	35	1538 (532)	1632 (565)	1775 (657)	1709 (582)	160 (53, 268)	0.004

^1^ Baseline deseasonalised 25(OH)D < 50 nmol/L.

**Table 3:** Forced vital capacity (mL) at baseline and follow-up (adjusted for age, sex, ethnicity and height) by treatment group.

Sample	*n*		Mean (Standard Deviation)				Change from Baseline, Vitamin D Minus Placebo	
	Vitamin D Group	Placebo Group	Vitamin D Group		Placebo Group			
			Baseline	Follow-UP	Baseline	Follow-Up	Mean (95% CI)	*p*-Value
Total	226	216	3078 (858)	3060 (861)	3107 (945)	3093 (910)	−5 (−49, 39)	0.83
Vitamin D-deficient ^1^	61	68	3098 (774)	3085 (783)	2961 (992)	2941 (949)	7 (−82, 95)	0.88
Asthma/COPD	54	59	2885 (833)	2863 (891)	2889 (976)	2868 (918)	0 (−96, 96)	0.99
Vitamin D-deficient ^1^ + asthma/COPD	16	27	2917 (757)	2967 (770)	2836 (1172)	2817 (1110)	69 (−109, 246)	0.44
Ever-smoker	104	113	2777 (846)	2979 (882)	3015 (901)	2976 (840)	42 (−19, 102)	0.17
Ever-smoker + vitamin D-deficient ^1^	26	28	3062 (805)	3091 (942)	2659 (780)	2573 (617)	115 (−14, 243)	0.08
Ever-smoker + asthma/COPD	25	35	2423 (718)	2486 (639)	2621 (840)	2598 (770)	86 (−52, 225)	0.22

^1^ Baseline deseasonalized 25(OH)D < 50 nmol/L.

**Table 4:** FEV1/FVC (%) at baseline and follow-up (adjusted for age, sex, ethnicity and height) by treatment group.

Sample	*n*		Mean (Standard Deviation)				Change from Baseline, Vitamin D Minus Placebo	
	Vitamin D Group	Placebo Group	Vitamin D Group		Placebo Group			
			Baseline	Follow-Up	Baseline	Follow-Up	Mean (95% CI)	*p*-Value
Total	226	216	76.4 (7.7)	76.0 (7.6)	76.6 (7.4)	75.4 (7.0)	0.7 (−0.1, 1.5)	0.07
Vitamin D-deficient ^1^	61	68	76.6 (6.5)	76.8 (7.0)	74.3 (8.6)	73.4 (8.0)	1.2 (−0.5, 2.9)	0.16
Asthma/COPD	54	59	65.9 (8.6)	66.1 (9.4)	68.0 (8.6)	66.8 (8.2)	1.4 (−0.2, 3.0)	0.08
Vitamin D-deficient ^1^ + asthma/COPD	16	27	69.7 (9.4)	70.2 (9.8)	67.3 (9.9)	66.0 (8.0)	1.9 (−0.8, 4.6)	0.16
Ever-smoker	104	113	75.2 (7.8)	75.0 (7.7)	75.3 (8.1)	74.0 (7.7)	1.1 (0.0, 2.5)	0.05
Ever-smoker + vitamin D-deficient ^1^	26	28	77.6 (6.0)	77.4 (6.6)	72.6 (11.0)	71.5 (8.8)	0.9 (−1.4, 3.2)	0.42
Ever-smoker + asthma/COPD	25	35	63.2 (8.2)	64.5 (10.5)	66.5 (9.6)	64.8 (8.8)	3.0 (0.7, 5.4)	0.01

FEV1 = forced expiratory flow in 1 s; FVC = forced vital capacity. ^1^ Baseline deseasonalized 25(OH)D < 50 nmol/L.

## Supplementary Materials

The following are available online at www.mdpi.com/2072-6643/9/12/1353/s1, Table S1: FEV1 *z*-scores at baseline and follow-up (adjusted for age, sex, ethnicity and height) by treatment group; Table S2: FVC *z*-scores at baseline and follow-up (adjusted for age, sex, ethnicity and height) by treatment group; Table S3: FEV1/FVC *z*-scores at baseline and follow-up (adjusted for age, sex, ethnicity and height) by treatment group; Table S4: Correlations of changes in observed 25(OH)D concentration with changes in lung function measures.
