# Supplementary material for: Effect of Monthly, High-Dose, Long-Term Vitamin D on Lung Function: A Randomized Controlled Trial
Source: Nutrients. 2017 Dec 13;9(12):1353. doi: 10.3390/nu9121353 (PMC5748803; doi:10.3390/nu9121353)
Supplement: Supplementary file 1 [file nutrients-09-01353-s001.pdf]

Table S1. FEV1 z-scores at baseline and follow-up (adjusted for age, sex, ethnicity and height) by treatment group

Sample	n		Mean (standard deviation)				Change from baseline, vitamin D minus placebo	
	Vitamin D group	Placebo group	Vitamin D group		Placebo group			
			Baseline	Follow-up	Baseline	Follow-up	Mean (95% CI)	P-value
Total	226	216	-0.73 (1.06)	-0.71 (1.06)	-0.68 (1.04)	-0.69 (1.01)	0.03 (-0.04, 0.11)	0.39
Vitamin D-deficient[1]	61	68	-0.54 (1.04)	-0.49 (1.05)	-0.96 (0.97)	-0.95 (0.97)	0.04 (-0.11, 0.20)	0.58
Asthma/COPD	54	59	-1.41 (1.12)	-1.37 (1.09)	-1.30 (0.92)	-1.33 (0.84)	0.06 (-0.11, 0.23)	0.46
Vitamin D-deficient[1] + asthma/COPD	16	27	-1.04 (1.30)	-0.84 (1.31)	-1.41 (1.04)	-1.45 (0.92)	0.24 (-0.05, 0.53)	0.10
Ever-smoker	104	113	-0.94 (1.14)	-0.88 (1.16)	-0.87 (1.06)	-0.94 (0.98)	0.13 (0.01, 0.24)	**0.03**
Ever-smoker + vitamin D-deficient[1]	26	28	-0.50 (1.06)	-0.40 (1.16)	-1.44 (0.95)	1.55 (0.82)	0.21 (-0.09, 0.51)	0.16
Ever-smoker + asthma/COPD	25	35	-1.94 (0.85)	-1.66 (0.87)	-1.39 (0.97)	-1.46 (0.84)	0.35 (0.11, 0.59)	**0.005**

[1]Baseline deseasonalized 25(OH)D<50 nmol/L.

Table S2. FVC z-scores at baseline and follow-up (adjusted for age, sex, ethnicity and height) by treatment group

Sample	n		Mean (standard deviation)				Change from baseline, vitamin D minus placebo	
	Vitamin D group	Placebo group	Vitamin D group		Placebo group			
			Baseline	Follow-up	Baseline	Follow-up	Mean (95% CI)	P-value
Total	226	216	-0.67 (0.99)	-0.63 (0.95)	-0.65 (1.01)	-0.60 (1.01)	-0.01 (-0.09, 0.07)	0.84
Vitamin D-deficient[1]	61	68	-0.44 (1.01)	-0.40 (0.98)	-0.82 (0.98)	-0.76 (0.95)	-0.01 (-0.20, 0.17)	0.87
Asthma/COPD	54	59	-0.77 (0.99)	-0.74 (0.89)	-0.84 (1.04)	-0.79 (0.97)	-0.02 (-0.21, 0.17)	0.83
Vitamin D-deficient[1] + asthma/COPD	16	27	-0.57 (1.06)	-0.37 (0.87)	-0.92 (1.06)	-0.86 (0.93)	0.15 (-0.21, 0.50)	0.42
Ever-smoker	104	113	-0.86 (1.01)	-0.79 (1.03)	-0.82 (1.11)	-0.82 (1.03)	0.08 (-0.03, 0.19)	0.17
Ever-smoker + vitamin D-deficient[1]	26	28	-0.42 (1.03)	-0.31 (1.08)	-1.24 (1.10)	-1.31 (0.90)	0.18 (-0.11, 0.46)	0.21
Ever-smoker + asthma/COPD	25	35	-1.66 (0.87)	-1.46 (0.70)	-1.34 (1.16)	-1.31 (1.04)	0.18 (-0.09, 0.45)	0.19

[1]Baseline deseasonalized 25(OH)D<50 nmol/L.

Table S3. FEV1/FVC z-scores at baseline and follow-up (adjusted for age, sex, ethnicity and height) by treatment group

Sample	n		Mean (standard deviation)				Change from baseline, vitamin D minus placebo	
	Vitamin D group	Placebo group	Vitamin D group		Placebo group			
			Baseline	Follow-up	Baseline	Follow-up	Mean (95% CI)	P-value
Total	226	216	-0.31 (1.07)	-0.35 (1.02)	-0.29 (0.97)	-0.41 (0.87)	0.08 (-0.03, 0.19)	0.13
Vitamin D-deficient[1]	61	68	-0.34 (0.91)	-0.29 (1.03)	-0.63 (1.06)	-0.73 (0.97)	0.15 (-0.09, 040)	0.22
Asthma/COPD	54	59	-1.75 (1.07)	-1.68 (1.13)	-1.47 (1.03)	-1.56 (0.95)	0.16 (-0.05, 0.37)	0.13
Vitamin D-deficient[1] + asthma/COPD	16	27	-1.23 (1.27)	-1.16 (1.38)	-1.56 (1.19)	-1.70 (0.88)	0.21 (-0.15, 0.57)	0.23
Ever-smoker	104	113	-0.44 (1.05)	-0.44 (0.97)	-0.41 (1.04)	-0.54 (0.95)	0.12 (-0.02, 0.26)	0.10
Ever-smoker + vitamin D-deficient[1]	26	28	-0.17 (0.76)	-0.20 (0.95)	-0.78 (1.38)	-0.89 (0.95)	0.07 (-0.24, 0.39)	0.63
Ever-smoker + asthma/COPD	25	35	-2.02 (1.00)	-1.79 (1.16)	-1.61 (1.15)	-1.75 (1.01)	0.37 (0.08, 0.65)	**0.01**

[1]Baseline deseasonalized 25(OH)D<50 nmol/L.

Table S4. Correlations of changes[1] in observed 25(OH)D concentration with changes[1] in lung function measures

Sample	Correlation coefficient (95% confidence interval)[3]		
	FEV1	FVC	FEV1/FVC
Total	0.03 (-0.06, 0.14)	-0.00 (-0.09, 0.09)	0.07 (-0.04, 0.16)
Vitamin D-deficient[2]	0.07 (-0.10, 0.24)	-0.02 (-0.22, 0.17)	0.16 (-0.04, 0.36)
Asthma/COPD	0.11 (-0.11, 0.34)	0.09 (-0.12, 0.29)	0.07 (-0.18, 0.30)
Vitamin D-deficient[2] + asthma/COPD	0.18 (-0.20, 0.55)	-0.00 (-0.46, 0.49)	**0.37 (0.05, 0.64)**
Ever-smoker	**0.17 (0.02, 0.31)**	0.10 (-0.05, 0.24)	**0.14 (0.01, 0.27)**
Ever-smoker + vitamin D-deficient[2]	**0.26 (0.01, 0.47)**	0.24 (-0.08, 0.50)	0.14 (-0.33, 0.50)
Ever-smoker + asthma/COPD	**0.34 (0.09, 0.55)**	**0.25 (0.00, 0.44)**	0.23 (-0.04, 0.46)

[1]Follow-up minus baseline.

[2]Baseline deseasonalized 25(OH)D<50 nmol/L.

[3]Adjusted for age, sex, ethnicity and height.
